# Sustainability and Financial Crime

**DOI:** 10.1007/s43576-022-00052-3

**Published:** 2022-03-23

**Authors:** Vincenzo Ruggiero

**Affiliations:** Middlesex University, The Burroughs, London, NW4 4BT UK

**Keywords:** Sustainability, Financial crime, Deregulation, Decriminalization

## Abstract

Sustainable development promises a wave of new approaches to environmental and social issues due to its perceived holistic nature. New ways of producing and consuming are purported to pave the way for smooth and consensual governance and the reduction of conflicts. The diversion of finance towards sustainable development may also impact on financial crime, at least in the views of optimists who focus on the connections between the two. It is felt that there is no really sustainable finance (the alignment of financial operations with sustainable development) without developing strong and efficient means to fight financial crime. This paper examines such optimistic views, providing, first, an account of institutional strategies relating to sustainable finance, and second, an analysis of some forms of financial crime. Focusing particularly but not exclusively on the UK, a final hypothesis is then formulated around the scenario we are likely to face in the near future if the financial sphere is coopted into the arena of sustainable development.

## Sustainable Development Goals

In March 2017, the UN listed seventeen Sustainable Goals to be pursued by member states. These included the end of poverty and hunger, the promotion of health and equitable education, gender equality, economic growth, crime reduction, access to affordable and sustainable energy for all, action against climate change, the promotion of peaceful societies, and the revitalization of global partnerships for sustainable development (UN, 2017). The last goal implied the participation of a key partner, namely the financial world, whose willingness to participate was deemed crucial for the partnerships to succeed. Concerns were initially expressed around the possibility of securing this key partner, as public funds were deemed inadequate and private investors were seen as reluctant to shift investments towards sustainable initiatives. Unlocking and reshaping finance became a priority for the attainment of the UN goals.

The initial concerns were compounded by the realization that environmentally degrading activities continued to dominate the economy, despite the increasing appeal of the concept of green finance.

In 2016, despite what had appeared as a positive momentum, the UN noted the accelerating decline in all major ecosystems and increasing economic inequality across the world, invoking more radical action from investors and, at the same time, urging the gradual abandonment of unsustainable economies and lifestyles.

‘Natural capital has declined in 116 out of 140 countries; 6.5 million premature deaths result from air pollution linked to the energy system; greenhouse gas emissions add energy to the Earth’s system at a rate equivalent to the detonation of four nuclear bombs every second; an average of 26.4 million people have been displaced from their homes by natural disasters every year since 2008, equivalent to one person every second’ (UNEP, 2016, p. 5).

Scholars identified obstacles and prompted policies to incentivize green investments, suggesting that more coordinated efforts could supersede the existing, fragmented and sectorial initiatives (Clark et al., 2018). More hopeful analysis saw, particularly in developing countries, a quiet revolution unfolding, announced by a new narrative making the matter of environment, climate and sustainable development the business of financial policy-makers and regulators (Zadek, 2018).

The extremely ambitious UN agenda requires an unprecedented mobilization of funds: some US$90 trillion over fifteen years. And while progress is currently recognized, further efforts are advocated that might create attractive enabling conditions for investors. Diverting public funds towards the private sector is one aspect of such efforts, which engage banks, stock exchange and insurance regulators. The G20 finance ministers and central bank governors have explored the possibility of embedding environmental considerations in financial activity and attempted to persuade stakeholders to invest in green development by reducing or eliminating risk. Persuasion also hinges on the argument that climate change will make the financial sphere increasingly unstable, prompting responses by all those operating in it (FSB, 2021). In brief, finance is as exposed to environmental degradation as every other collective or individual actor, hence the need for it to mobilize alongside everybody else.

## Growing Turmoil and Uncertainty

Lack of sustained and adequate response from the financial sector could be ascribed to two factors among others.

First, the current routine involvement of finance in the funding of environmentally degrading operations, particularly in developing countries. Despite suggestions that companies should link financial performance to environmental concerns, no clear business case is being made for broader issues of sustainability (Epstein & Roy, 2003; Yasin et al., 2021). Finance, therefore, may become co-perpetrator of environmental crime and even complicit in violent responses to campaigners who attempt to defend territories from corporations pursuing oil, gas and minerals (Ruggiero, 2020).

Second, inadequate response from the financial sector can be ascribed to the general tendency of business to prioritize immediate goals. Political instability and economic volatility in most countries may account for investors’ reluctance to engage in long-term initiatives whose outcomes are perceived as uncertain. This is a constant feature of the context in which enterprises operate, although economic history shows that uncertainty itself plays a crucial role in mobilizing activity, defying risk and modeling innovation. Turmoil creates challenges and paves the way for unpredicted advantages.

It is perhaps with this in mind that the UN Environmental Programme encourages transformation in the financial sphere by leveraging the current uncertainty. Financial innovation, in this sense, is seen as necessary due to the ongoing turmoil, which is bound to deliver a new philosophy of development. Systematic national action, public–private coalitions and market innovation are the suggested strategies, inscribed in a cooperative framework that rejects decisions made in separate silos. Cooperation is aimed at combining the strength of diverse actors historically inured to independent action. To this end, a five-step process is described that is expected to embed finance at the heart of sustainable development. The five steps, described as Accelerators of Transformative Finance, are:

1. National financial market reform geared to the UN Sustainable Development Goals and the Paris Agreement on climate change.
2. New technologies applied to financial activity, particularly in developing countries.
3. Redeployment of public finance.
4. Investments in awareness-raising so that the financial community can implement new approaches and plans.
5. Development of common methods, tools and standards that allow for the measurement of sustainable priorities.

While some of these measures are being perfected and timidly implemented, events seem to demonstrate that the financial sphere remains for now unperturbed. Rather than follow a sustainable path, financiers keep operating in conventional areas where uncertainty about profits is limited. Global Witness (2021), for instance, revealed how state and private financial groups keep funding firms that pursue destructive rather than green growth, particularly in programmes of deforestation. In response, national and international agencies are striving to reduce uncertainty in new sustainable areas through the identification of specific, safe areas of investment. In an effort to encourage investors to integrate climate change and broader sustainability concerns in their financial choices, a number of jurisdictions have elaborated typologies and taxonomies of sustainable finance (OECD, 2021). These provide a comprehensive classification of which investments are green and sustainable, which are less risky and more remunerative, which are less hampered by red tape and regulation. Market clarity and improved confidence are the goals of such initiatives, which include systems for the tracking of sustainable finance flows and the assessment of their effectiveness. Strangely, the aviation and health sectors are not covered.

Nuanced typologies have been adopted in the EU, China, Japan, France and the Netherlands, while other countries expressing an interest in sustainable finance taxonomies include Canada, Kazakhstan and Indonesia. Some activities are labeled ‘not compatible with environmental objectives’, others as ‘not significantly harmful’, while yet others as ‘light green’ or ‘on a transition pathway to become green’. Consideration is given to restraints brought by the Covid-19 crisis, which is overstretching human and financial resources. Such restraints are seen as an obstacle for investors to try new ventures in relatively unknown areas.

Taxonomies, it is to be expected, will turn into new rules, and it would be too harsh to infer that uncertainty and the above-mentioned restraints will lead to tolerance towards deviations from the new rules. On the other hand, tolerance towards investors punctuates the history of the financial world and characterizes the most recent events that occurred in that world. Tolerance may take the form of ‘nudging’, as proposed by behavioral economists who explain how choices can be guided by the opening of new opportunities or the abolition of old prohibitions. A persuasive form of nudging financial operators consists of displaying tolerance in the face of their constant search for innovative practices, including those of a deviant nature. Focusing on tax havens may provide a significant backdrop against which tolerance and innovation in the financial sphere may develop.

## Avoidance and Evasion

Tax havens offer services to a variety of individuals and groups engaged in distinct operation, namely tax avoidance, tax evasion and money laundering.

Major beneficiaries of tax avoidance are corporations trading in one high-tax jurisdiction and paying their dues in low- or zero-tax havens. Despite the manifest, and to some ‘outrageous’, unfairness that permits large firms to pay less tax than small struggling businesses, tax avoidance is a legitimate, if unethical, conduct. It is proof, as conflict theorists in criminology would have it, that law serves the interests of some social groups rather than society at large.

Tax evasion is illegal and consists in violations that, predominantly, benefit those who already benefit from existing laws. It is, therefore, crime mainly committed by privileged individuals and groups against a system that guarantees their privileges. It is enacted through turning legally acquired money into illegal funds (Ruggiero, 2017a, 2017b).

Money laundering does exactly the opposite: it turns illegally earned money into apparently legal profits. This specific form of financial crime, therefore, is deemed an appendix of organized crime itself, namely an auxiliary, if integral, component of illicit markets.

Tax havens are not only located in distant, exotic, zero-tax islands, but are also situated at the very heart of financial centers. The UK is certainly not a zero-tax country, at least not for all. This notwithstanding, it is regarded as one of the major final destinations for hidden funds from across the world. This is due to its reputation of respectability and professionalism, and to the discretion with which it offers its services. The variety of expert and skilled agents populating the City of London, including lawyers, accountants, advisors, mediators and fiduciaries, can cater for a diverse clientele, from members of organized criminal groups to corrupt politicians and entrepreneurs. Funds derive from drug trafficking, illegal trade in arms, people smuggling, stolen art work, protection rackets and all other activities in which organized crime is engaged. This was also the opinion, expressed in 2015, by the head of the National Crime Agency (NCA) Keith Bristow, who warned that Britain’s economy, the security of its financial sector and its reputation for probity were in jeopardy because of the emergence of London as a world center for criminal financial activity. Profits from crime linked to international terrorism were also said to find hospitality in London. The revelations were accompanied by the decision to investigate law firms for their role in money laundering and other forms of economic crime by large-scale criminal organizations (NCA, 2015).

The City of London, however, is not alone in providing such services, as it competes with financial centers such as New York and Tokyo. All three centers engage in ‘off-shore activities’, a term that, as already noted, does not refer to the geographical locations in which certain financial activities are carried out, but to the judicial status of the financial centers themselves (Talani, 2011). The Crown Dependencies and the British overseas territories are components of the tax haven and money laundering network, as they enjoy autonomy in the sphere of taxation and discretion in financial issues (Palan, 2003).

This large network encapsulates a significant part of irregularly circulating money and the hidden wealth of nations, while keeping the City of London dissociated from the unorthodox business committed elsewhere on its behalf. Various other havens such as Hong Kong, Luxembourg and Switzerland, while not British, still feed large volumes of business to the City.

Collectively, these secrecy jurisdictions act as hidden conduits for dirty money originating from countries across the world, and when reaching London the origins of this money can be untraceable, being hidden behind a complex of secretive offshore bank accounts, companies and trusts (Christensen, 2015).

Despite the growing evidence that details specific activities taking place in tax havens, there is a sense that leading economies will keep refusing to address the problem at its source. The UK overseas territories and crown dependencies, it is still felt, cannot be shut at a stroke, because the official banking system thrives on the benefits of offshore centers and tax havens.

In brief, it should be emphasized that money from organized criminal activity, bribes and tax evasion flows in the same pool, in networks that gather individuals from a variety of social and occupational backgrounds. Actors operating in them are socially ‘fuzzy’, in the sense that their exploits and careers overlap with those of others who are apparently radically different from them. Financial networks are the reflection of grey areas hosting diverse cultures, identities and motivations, areas in which disparate activities develop points of contact, common interests and strategies between licit, semi-licit and overtly illicit economies. These are ‘dirty economies’ consisting of encounters which add to the respective cultural, social and symbolic capital possessed by criminals, politicians and entrepreneurs, who interlock their practices.

A brief look at the Panama Papers, the Paradise Papers and the most recent Pandora Papers might clarify the trajectory of institutional tolerance vis-à-vis deviant financial action. The three cases sketched below illustrate the links, as initially postulated, between sustainable finance and financial crime.

## Panama–Paradise–Pandora

### Case 1

The leaking of millions of documents revealed details of operations conducted over forty years in offshore tax havens. The Panamanian law firm Mossack Fonseca was at the center of the revelations, which provided information about some seventy current or former heads of state and their tax evasion. Iceland’s Prime Minister resigned, while Vladimir Putin’s circle was proven to engineer unorthodox wealth-acquisition mechanisms. Representatives of FIFA (the International Football Federation) were also listed in the documents. Mossack Fonseca, initially, claimed to be shocked by the way the services offered had been abused by customers, but was also surprised that offshoring arrangements were so vulnerable to investigative journalism and, perhaps, to prosecution (Ruggiero, 2017a). British and London-based banks emerged among the most active customers of the Panama firm: HSBC, Coutts, Rothschild and UBS being among the top ten banks who set up around 15,600 shell companies to help clients conceal their finances. HSBC had been fined £28 million in 2015 for allowing customers to launder money in its Swiss private branch, while in Panama it set up more than 2,300 offshore companies. Its chief executive was among the customers of Mossack Fonseca, which concealed his pay and dealt with his tax affairs. Coutts, the private arm of publicly owned Royal Bank of Scotland, set up 500 paper companies through its Jersey agency. UBS, the Swiss group with most of its investment banking operations in the City of London, set up more than 1300 offshore companies. The Luxembourg International Bank was involved through Experta, which offers corporate and trust services, while other British-based institutions included Credit Swiss Channel Island and Rothschild Trust Guernsey (Goodway, 2016).

The Panamanian firm also laundered money derived from notorious bank robberies and other organized criminal activities. Fonseca’s customers avoided paying tax by hiring Bahamas residents as fronts. UK Prime Minister David Cameron and his father Ian appeared in the leaked papers, along with six members of the House of Lords, three former Conservative Members of Parliament and dozens of donors to UK parties. Ian Cameron was the director of an investment fund named (how ironic!) *Blair*more Holding Inc, which had among its customers an adviser of Robert Maxwell and the Rolling Stones. In thirty years, Blairmore never paid a penny of tax in the UK on its profits (Garside, 2016).

The British Virgin Islands continued to licence Mossack Fonseca despite knowing the firm was unable to establish who owned the companies on its books, while a British banker set up a secret offshore finance company allegedly used by North Korean leaders to assist in arms sales and the expansion of its nuclear weapon program. The revelations also touched Ukraine’s president Poroshenko, who was elected in 2014, in the aftermath of the political upheaval in the country that led to the annexation of Crimea and open conflict with Russia. While the war was taking place, Poroshenko moved his assets into an offshore company in the British Virgin Islands. The leaks also contained information about how some leaders from a number of countries used foundations and other firms registered in Panama to anonymously own mining companies and real estate (Schmidt & Lee Myers, 2016).

### Case 2

One of the oldest banks in Central America was established in 1902 in what was then British Honduras, now known as Belize. The bank operated as a branch of the Royal Bank of Canada and traded extensively across the Caribbean region. In 1987, Belize Bank International (BBI) was bought by Michael Ashcroft, the son of a British colonial administrator, who advised the local government on how to turn the country into an offshore financial center. After being granted a thirty-year special tax break, the bank attracted liquidity from all over the world (Bowers, 2016; Brooks, 2016). US authorities are still unable to ascertain whether the bank was, and still is, used by US citizens to hide assets and evade tax. Nor can the bank be forced to disclose the identity of all its clients, irrespective of nationality. Like all offshore banks, BBI operates through correspondent financial institutions, based in the USA and elsewhere, that provide services and process transactions. If the accounts in correspondent institutions are shut off, the offshore bank is out of business and all customers’ deposits are frozen. Among the correspondent institutions linked to BBI are Citibank and Bank of America. According to the IMF (International Monetary Fund), the investigation resulted in the loss of correspondent banking relationships, causing destabilizing effects on the financial and economic stability in the Caribbean region. In response, Prime Minister of Belize, Dean Barrow, proposed that the region could establish its own banks in the USA and establish correspondent relationships with them. Perhaps Mr Barrow ignored the de-risking strategies designed by financial institutions that deny services to ‘risky’ customers. Or perhaps he deemed such strategies ineffective. He must have also considered that the last 30 years of money laundering control achieved very little. Alternatively, the banks of the region, through mergers, would have to achieve a critical mass to make the Caribbean’s banking industry more attractive to financial institutions in the USA and across the world (Ramos, 2016). This investigation was highly embarrassing for the UK government as the bank is owned by a former deputy chair of the Conservative party and one of its most generous donors. Investigators based their work on a voluminous file composed over the last ten years which lists, among the bank’s customers, tax dodgers and members of transnational criminal organizations (ibid).

### Case 3

In October 2021, a leak revealed the deals of thirty-five world leaders, 300 officials and 130 billionaires. Offshore financial data exposed the reliance of the UK Conservative Party on a narrow group of extremely influential donors. Emails, share certificates and secretive contracts showed how lobbying and donations proceeded in tandem, as the cases involving Swedish Telecom and Russian company Aquind, the latter seeking UK governmental approval to build a power interconnector under the Channel. The wealth hidden by oligarchs and government minsters from several countries emerged, but all denied wrongdoing. The papers highlighted that ‘there is nothing inherently illegal about in the setting up of tax haven structure. Examples include the Czech prime minister using a complex offshore chain to buy a £13m mansion in the south of France. Cyprus’s president, through the law firm that bears his name, helped conceal the assets of tycoons behind the name of fictitious owners. Ukraine’s president was named in connection with a network of offshore companies and undeclared firms operating in the British Virgin Islands. Some of the politicians owned luxury properties. The Guardian noted that despite all the disclosures and all promises from politicians about closing loopholes and making things more transparent, the rich and influential continue to hide assets, paying little or no tax (Harding, 2021).

These cases demonstrate how colonial and postcolonial links still prevail in the financial as well as in other spheres, as unorthodox operations carried out in distant exotic regions, in reality, attend to the interests of global elites located in the most developed area of the earth. However, as already noted, tax havens are also situated at the very heart of financial centers, although the stigma for illicit practices regularly falls on peripheral world regions connected to them. Focusing on the UK, it has been suggested that, after the demise of empire, a network of secrecy jurisdictions were created, linked to the City of London financial interests. It is estimated that today up to half of global offshore wealth is hidden in British jurisdictions and that Britain and its dependencies are the largest global players in the world of international finance (Oswald, 2018).

The last case examined brings to the fore the ambiguity of some conducts in the financial sphere, to the point that the criminal label appears to tone down, to fade away, as is often the case with a variety of white-collar offences. This process leads straight to Edwin Sutherland and his groundbreaking work produced almost a century ago.

## Is Financial Crime Crime?

When Sutherland examined crimes committed by large corporations, he posed the rhetorical question ‘Is white-collar crime crime?’ Long before contemporary formulations that replace the study of crime with that of harm, he intended to highlight how certain socially damaging conducts were excluded from the criminal justice arena. Perhaps, he also imagined that his efforts would lead to the criminalization of powerful offenders. On the contrary, what we see in the three cases presented above is a process of legalization of certain conducts, which may be ethically stigmatized but remain juridically unassailable.

It is worth recalling that the Panama Papers scandal (case 1) erupted in the aftermath of the 2008–10 financial crisis, when responses by national and international agencies appeared to be robust enough to prevent unashamed and obvious irregularities. However, the measures proposed to prevent future crises were contested, amended or scrapped. When applied, their potential effect was neutralized through the creation or expansion of areas impervious to regulation. The phenomenon possesses some similitude with illegal drugs markets, where enforcement targeting one substance or distributing route directs business towards other substances and routes, like a balloon ‘bulging’ here or there according to where it is squeezed.

The ineffectiveness of new regulations was also the result of the lack of substantial organized and ideological opposition to market philosophies, whereby policies continued to be tailored around the needs of bankers rather than citizens.

With the Paradise Paper (case 2) new aspects of deregulation came to the surface in the form of tax breaks: the BBI was able to attract customers from all over the world. Hiding money, in this way, became a decriminalized act. The decriminalization process finds its apt conclusion with the Pandora Papers (case 3), when all accounts of the ‘scandal’ feel the necessity to warn that the conducts reported were totally legal. For instance, after stating that Conservative party’s donors were all enmeshed in the secret offshore world, journalists could only ponder on the implications for political funding rather than on its harmful or criminal nature. Reporting about Tony and Cherie Blair saving £300,000 in stamp duty when they bought a London office via an offshore company, they found it appropriate to add that the operation was entirely legal. Documents proving that Mohamed Amersi, a major Tory backer, had acted as a mediator for a Swedish telecoms deal are devoid of any indication why the deal was corrupt. Similarly, where the files feature a former Russian government minister who gifted over £2 m to the Tories and spent £160,000 on a game of tennis with Boris Johnson, comments revolve around mansions, yachts and swimming pools, signaling moral indignation not condemnation of criminal behaviour. True, all the items that shape luxurious lifestyles are paid for via offshore accounts, but the mantra goes: there is nothing inherently illegal about setting up a tax haven structure.

The three cases discussed would suggest that global finance is no longer subject to political control; on the contrary, it is politics that has placed itself at its service. The trend that appears to emerge amounts to a shift in institutional responses to financial irregularities. More details about the **P triad** may add further light to this process.

With the Panama Papers, criminal probes were launched into tax evaders and their lawyers. Charges included fraud and conspiracy to commit money laundering. Many of the individuals exposed of wrongdoing were held to account and in many cases forced to resign from their various posts, including the Prime Minister of Iceland. Some of the people involved approached the tax authorities to settle their affairs before action against them was taken. Arrest orders were issued, although they were nullified by Panama’s constitution that prohibits extradition of its citizens (Zamorano, 2020). The International Consortium of Investigative Journalists ‘celebrated’ its success by following offenders who moved ‘from front pages to prison time’ (Fitzgibbon, 2021). Even conservative papers had to admit that the Panama Papers showed the vulnerability of money laundering controls (The Times, 22 June 2018).

With the Paradise Papers money launderers and tax evaders became less newsworthy than Appleby, the Bermudian law firm from which leaked information originated. Appleby issued legal proceedings against the BBC for breach of confidence, while multinational Glencore sought an injunction to prevent the use of the leaked documents by tax authorities (Byrne, 2019). Litigation brought by Appleby against the BBC and The Guardian was settled after it became clear that the documents were no longer owned by the law firm and were not legally privileged (Appleby, 2018).

Commentators of the Pandora Papers laid bear the global entanglement of political power and secretive offshore finance, but abstained from labeling such partnerships as criminal. The general view was that the documents unveiled created a new momentum for ending the abuse of corporate secrecy and pushing decision-makers into action. In the UK a petition urged ‘to review the tax breaks for corporate and wealthy individuals to increase the resources of the HMRC (Her Majesty Revenue & Customs), to enforce tax legislation and tighten the regulations of accountants, lawyers and bankers’ (You 38 Degrees, 2020). Nine countries launched investigations into the Pandora Papers revelations: India, Pakistan, Mexico, Spain, Brazil, Sri Lanka, Australia, Panama and the Czech republic. In the UK, the Tories were just asked to return the cash from donors named in the revelations. Progressives called for a rapid measure that would solve the problem once and for all: abolish anonymous companies by passing legislation that the true owner of companies operating in the country must declare themselves (Siddique & Weaver, 2021).

Echoing a religious creed, the prevailing motto is: there is no salvation outside the market (Todorov, 2014). Against this ideological backdrop, licit or illicit financial operations, both causing social harm, may be destined to continue undeterred as long as those conducting them can claim that such operations benefit not themselves, but markets, namely society at large. In this way, as Touraine (2014: 74) has remarked, financiers can step outside the framework of legality and enter the world inhabited by ‘drug cartels, arms dealers or cigarette smugglers’, while their acts become ‘part of the powerful surge in an expanding illegal economy’. In the grey areas of such economy, white-collar crime joins and overlaps with conventional forms of organized criminality, shaping ‘networks of greed’ formed of individuals and groups from diverse social backgrounds and subcultures. Such grey areas thrive irrespective of booms and slumps and contain money laundering, tax evasion and bribes.

Calls for closing what are described as ‘loopholes’ miss the mark, as they do not grasp their latent function. What appear to be loopholes, namely normative imperfections subject to abuse, are in fact ‘nudges’ that make financial operations less uncertain and guide operators towards a reassuring future. Similar nudges emerged when EU finance ministers meeting in Luxembourg decided to reduce the number of countries on a tax haven blacklist. Anguilla, Dominica and Seychelles were removed. The British Virgin Islands was not even on the list, despite accounting for two-thirds of the shell companies in the Pandora files.

The British government is reluctant to implement offshore reform, as already remarked, for fear of revealing the identities of those who use offshore companies to buy UK property. There is no public register for property so bought. Which means London remains a highly attractive destination for kings and kleptocrats. The king of Jordan is said to have amassed a $100m property portfolio that includes three buildings in Belgravia (Goodley & Safi, 2021). Money from organized crime and corruption rubs shoulders with funds from those who merely seek to evade public scrutiny.

Nudges are also found in the area of regulation, an area accused of failing from top to bottom. Regulators could not prevent financial organizations from paying out bonuses of more than £125m to staff since 2016 (Ungoed-Thomas, 2021). In 2021 these were among the biggest bonus pots ever handed out in a government department or quango. Gina Miller, the business activist and co-founder of the True and Fair Campaign, said the UK has a regulatory body which is not fit for purpose: ‘We have seen over the last five years some of the biggest financial scandals due to a lack of enforcement and regulatory rigor. It’s unbelievable against that backdrop to award these bonuses’ (ibid).

In its turn, the Financial Conduct Authority (FCA) in the UK is now proposing to scrap the bonuses after independent reviews found the regulator had acted too slowly to protect customers. Moreover, bonuses had not been effective at driving individuals or collective performance. The FCA is funded by the financial firms it regulates and employs some 4,200 staff. It was criticized in a damning report by the former court of appeal judge Dame Elizabeth Gloster over its failure to effectively supervise and regulate the mini-bond issuer London Capital & Finance (LCF). About 11,600 investors lost savings of up to £237m when LCF went into administration in 2019. The watchdog also failed to intervene before the collapse of Neil Woodford’s £3.1bn Woodford Equity Income Fund, which was shut down in October 2019 with heavy losses of tens of thousands of investors (Ungoed-Thomas, 2021).

Finally, asset recovery of evaded taxes has proven totally ineffective. In the UK bribery and corruption activities appear to be a very low priority, with no recoveries at all made until 2011–12, and overall recoveries from bribery-corruption just 0.2 per cent of total recoveries in 2003–13. The recovery agencies appear, from the data available, to focus upon easy target, such as perpetrators of benefit fraud.

## Sustainable Finance with Benefits

It would appear, therefore, that the operational field of finance is being expanded and cleared of legal obstacles. The situation thus created could be described as one conducive to a form of primitive accumulation that will engender the desire to invest into novel areas, including green initiatives. With the reduction of uncertainty and the reassurance that investors will be unlikely to be called to account for the practices they choose, sustainable finance will not reduce financial crime, but will be boosted by its decriminalization.

This process has been hatched during years of financial ‘innovation’, a noun that hides less flattering meanings. According to Krugman (2020), financial innovation amounted to finding ways to evade regulation, after market players became horrified by the sudden realization that they did not understand the complex system they had created. The ensuing collapse of trust turned into operators growing reluctant to lend money to each other, as they were unsure they would be repaid. As things became increasingly opaque, ‘innovation’ was the answer, a word ‘that should from now on strike fear into investors’ hearts’ (ibid: 90). Opacity and confusion, in brief, led to deregulation that made investments safer, namely placing them beyond the reach of the law.

Panama, Paradise and Pandora describe a process leading to the convergence of tax avoidance and tax evasion. The former, typically committed by corporations and the wealthy, was ethically condemned if legally accepted, but was also deemed a lesser evil than tax evasion. Those who avoided taxation were regarded as unlikely to hide money from the tax authority, a practice that the privileged were supposed to find unnecessary. Instead, the privilege of avoiding tax morphed into opportunities to join the pool of evaders, with the consequence that ‘the truly wealthy end up paying a much lower effective tax than the merely rich, not because of loopholes in tax law, but because they break the law’ (ibid: 349).

The benefits sustainable finance may enjoy remind us of the notion of sabotage elaborated by Veblen (1982, 1994). Associated with the weakening of the productive capacity of systems, sabotage was the name given by Veblen to entrepreneurs who denigrated manufacturing, who encouraged wasteful consumer preference rather than produce serviceable goods, responsive to real needs (Appiah, 2021; Camic, 2020). The concept of sabotage, here, could be described not only as an implicit affirmation of the priority of financial over productive activity, but also as an entitlement by operators to constantly experiment with novel practices. In other words, a new version of Veblen’s sabotage is to be found in a series of unorthodox conducts aimed at empowering operators to the point of making them ‘too big to fail’. What is pursued is a form of silent guarantee that representative institutions will rescue finance irrespective of its choices and their social impact. Financial operators, consequently, will continue experimenting, fully aware that their recklessness will be bailed out by taxpayers (Nesvetailova & Palan, 2013, 2020). Officially faithful to the sacred values of competition, they have devised an impressive array of techniques that deny those values.

As Veblen intuited, rational, self-interested individuals operating in the economy are guided by the public institutions they pretend to abhor. They require that such institutions limit their role to the protection of the lives and wealth individuals create for themselves. At the same time, rather than competing in markets, they compete in the race to get as close as possible to the sources of public wealth.

Persuading finance to invest in sustainable development means encouraging operators to participate in this race. It also means creating narratives and slogans that accompany the economic change being pursued (Shiller, 2020). Popular wisdom will have to be mobilized and spread, creating what narrative economics terms a contagious situation that pushes people towards certain goals and the acceptance of the means to achieve them. Boris Johnson’s narrative limits itself to the replacement of ‘greed’ with ‘green’, rightly implying that business will hardly be involved if not through the promise of new, unprecedented profits: ‘green is good’, he told Bill Gates (Hughes, 2021).

## Conclusion

The United Nations Environment Programme (UNEP, 2016) stresses that aligning the financial system with sustainable development is ultimately a policy choice, involving governments, central banks, financial firms and regulators. Such choices have already been made with the 2030 Agenda and the 2016 Paris Agreement, although the goals identified are not yet embedded across the financial system and the capital flows are not yet consistent with sustainable development.

In the previous pages, after an overview of the UN Sustainable Development Goals, the variable uncertainty has been examined, namely what allegedly constitutes a key hurdle that diverts financial flows from sustainable investments. However, as a constant feature of markets in general and financial markets in particular, uncertainty has also been identified as a propellant for new entrepreneurial exploration, a powerful reagent for innovation. A key variable for the analysis of the enterprise, innovation refers to the search for new raw materials, new labor arrangements, new productive strategies and new markets, designating, in Schumpeterian terms, the genuine evolutionary nature of the economy engaged in a constant fight against stagnation. An account of the Panama, Paradise and Pandora Papers shows how innovation in the financial world is connected to deregulation, namely a process that demolishes legal obstacles and directs investments towards novel areas. From this perspective, it has been argued that sustainable finance (the alignment of financial operations with sustainable development) is unlikely to prevent financial crime, on the contrary, it will more probably induce the legalization of this type of criminality.

In conclusion, a paradoxical scenario is facing us: sustainable finance will grow increasingly unsustainable due to its hypertrophic growth beyond the boundaries of the law. In such a scenario, political choices will be hampered by the very withering of politics, destined to become a subsystem subsumed by the economy.

Some prevalent policies addressed to the unemployed rely on the belief that those out of work should not be financially assisted lest they are encouraged to remain idle. Policies addressed to the financial operators take the opposite stance, as they push into hyperactivity through the provision of benefits that transcend the legitimate sphere.
